# Supplementary material for: Multi‐dataset identification of innovative feature genes and molecular mechanisms in keratoconus
Source: J Cell Mol Med. 2024 Sep 19;28(18):e70079. doi: 10.1111/jcmm.70079 (PMC11412914; doi:10.1111/jcmm.70079)
Supplement: Supplementary file 2 — Material S2. [file JCMM-28-e70079-s002.zip › 7.GSEA analysis/6.SNORA74B/KEGG_INOSITOL_PHOSPHATE_METABOLISM.html]

Details for gene set KEGG\_INOSITOL\_PHOSPHATE\_METABOLISM[GSEA]

|  || Dataset | SNORA74B.group.cls#h\_versus\_l |
| Phenotype | group.cls#h\_versus\_l |
| Upregulated in class | l |
| GeneSet | KEGG\_INOSITOL\_PHOSPHATE\_METABOLISM |
| Enrichment Score (ES) | -0.4381152 |
| Normalized Enrichment Score (NES) | -1.6983521 |
| Nominal p-value | 0.01010101 |
| FDR q-value | 0.31123495 |
| FWER p-Value | 0.387 |
Table: GSEA Results Summary

  

Fig 1: Enrichment plot: KEGG\_INOSITOL\_PHOSPHATE\_METABOLISM      
 Profile of the Running ES Score & Positions of GeneSet Members on the Rank Ordered List

  

| SYMBOL | TITLE | RANK IN GENE LIST | RANK METRIC SCORE | RUNNING ES | CORE ENRICHMENT || 1 | INPP4B | na | 2598 | 0.326 | -0.0322 | No |
| 2 | SYNJ1 | na | 7655 | 0.145 | -0.1590 | No |
| 3 | PLCG2 | na | 8239 | 0.131 | -0.1588 | No |
| 4 | MINPP1 | na | 8901 | 0.116 | -0.1626 | No |
| 5 | PLCB3 | na | 9334 | 0.106 | -0.1611 | No |
| 6 | IPMK | na | 9557 | 0.101 | -0.1543 | No |
| 7 | PLCB2 | na | 9573 | 0.101 | -0.1414 | No |
| 8 | PLCG1 | na | 9672 | 0.099 | -0.1313 | No |
| 9 | PIKFYVE | na | 10605 | 0.081 | -0.1475 | No |
| 10 | INPP1 | na | 10748 | 0.079 | -0.1412 | No |
| 11 | IPPK | na | 11091 | 0.073 | -0.1415 | No |
| 12 | PIP4K2B | na | 11358 | 0.069 | -0.1401 | No |
| 13 | PLCD3 | na | 12496 | 0.053 | -0.1659 | No |
| 14 | PTEN | na | 12756 | 0.050 | -0.1668 | No |
| 15 | PIK3CA | na | 13079 | 0.046 | -0.1701 | No |
| 16 | PLCD1 | na | 13448 | 0.042 | -0.1752 | No |
| 17 | INPP5J | na | 14023 | 0.035 | -0.1871 | No |
| 18 | PIK3CB | na | 15769 | 0.017 | -0.2352 | No |
| 19 | INPP5K | na | 15823 | 0.016 | -0.2346 | No |
| 20 | PLCB4 | na | 16838 | 0.006 | -0.2630 | No |
| 21 | ALDH6A1 | na | 16860 | 0.006 | -0.2628 | No |
| 22 | PIK3C2B | na | 16971 | 0.005 | -0.2654 | No |
| 23 | INPP5E | na | 18387 | -0.011 | -0.3048 | No |
| 24 | PIP5K1C | na | 18480 | -0.012 | -0.3059 | No |
| 25 | PIK3CG | na | 18884 | -0.016 | -0.3155 | No |
| 26 | IMPA1 | na | 19024 | -0.017 | -0.3172 | No |
| 27 | PLCZ1 | na | 19726 | -0.026 | -0.3341 | No |
| 28 | PIK3C2A | na | 20182 | -0.030 | -0.3432 | No |
| 29 | INPPL1 | na | 20210 | -0.031 | -0.3400 | No |
| 30 | PIK3CD | na | 20354 | -0.032 | -0.3399 | No |
| 31 | TPI1 | na | 20621 | -0.035 | -0.3430 | No |
| 32 | INPP5A | na | 21505 | -0.046 | -0.3624 | No |
| 33 | PIK3C3 | na | 22677 | -0.061 | -0.3882 | No |
| 34 | CDIPT | na | 24408 | -0.088 | -0.4266 | Yes |
| 35 | SYNJ2 | na | 24669 | -0.092 | -0.4220 | Yes |
| 36 | PI4KB | na | 25039 | -0.099 | -0.4197 | Yes |
| 37 | OCRL | na | 25177 | -0.102 | -0.4103 | Yes |
| 38 | PIP4K2C | na | 25967 | -0.116 | -0.4178 | Yes |
| 39 | INPP4A | na | 26031 | -0.117 | -0.4042 | Yes |
| 40 | PLCD4 | na | 26111 | -0.119 | -0.3909 | Yes |
| 41 | PI4KA | na | 26267 | -0.122 | -0.3794 | Yes |
| 42 | ITPKA | na | 26318 | -0.123 | -0.3646 | Yes |
| 43 | ITPK1 | na | 26769 | -0.134 | -0.3600 | Yes |
| 44 | PIP4K2A | na | 26936 | -0.138 | -0.3467 | Yes |
| 45 | IMPA2 | na | 27158 | -0.143 | -0.3343 | Yes |
| 46 | INPP5B | na | 27989 | -0.164 | -0.3367 | Yes |
| 47 | MIOX | na | 28180 | -0.170 | -0.3200 | Yes |
| 48 | PIK3C2G | na | 28884 | -0.191 | -0.3152 | Yes |
| 49 | ITPKB | na | 30431 | -0.243 | -0.3280 | Yes |
| 50 | ISYNA1 | na | 31897 | -0.317 | -0.3287 | Yes |
| 51 | PLCB1 | na | 32854 | -0.381 | -0.3064 | Yes |
| 52 | PIP5K1A | na | 33089 | -0.403 | -0.2603 | Yes |
| 53 | PLCE1 | na | 34694 | -0.990 | -0.1768 | Yes |
| 54 | PIP5K1B | na | 34724 | -1.356 | 0.0001 | Yes |
Table: GSEA details [plain text format]

  

Fig 2: KEGG\_INOSITOL\_PHOSPHATE\_METABOLISM      
 Blue-Pink O' Gram in the Space of the Analyzed GeneSet

  

Fig 3: KEGG\_INOSITOL\_PHOSPHATE\_METABOLISM: Random ES distribution      
 Gene set null distribution of ES for **KEGG\_INOSITOL\_PHOSPHATE\_METABOLISM**
